# Bridge the GAPPS (gastric adenocarcinoma and proximal polyposis syndrome)

**DOI:** 10.1016/j.igie.2025.06.001

**Published:** 2025-06-10

**Authors:** Suchapa Arayakarnkul, David Brauer, Christopher J. LaRocca, Daniela Guerrero Vinsard

**Affiliations:** 1 Department of Medicine, University of Minnesota, Minneapolis, Minnesota, USA; 2 Department of Surgery, University of Minnesota, Minneapolis, Minnesota, USA; 3 Division of Surgical Oncology, Department of Surgery, University of Minnesota, Minneapolis, Minnesota, USA; 4 Division of Gastroenterology and Hepatology, Minneapolis VA Medical Center, Minneapolis, Minnesota, USA; 5 Division of Gastroenterology, Hepatology and Nutrition, University of Minnesota, Minneapolis, Minnesota, USA

A 46-year-old woman with an extensive family history of gastric adenocarcinoma (2 maternal aunts and 1 cousin) presented with a 2-year history of abdominal pain and nausea. Esophagogastroduodenoscopy (EGD) revealed innumerable medium-sized sessile polyps in the gastric fundus with complete antral and small bowel sparing (Fig. 1A and B). Sampling of multiple polyps was performed by taking biopsy specimens with a cold forceps, and the biopsies revealed fundic gland polyps (FGPs) without dysplasia. Colonoscopy and abdominal computed tomography imaging were normal. Genetic testing was positive for a point mutation c.-30417T>C in the promoter 1B region of the *adenomatous polyposis coli* (*APC*) gene. A diagnosis of gastric adenocarcinoma and proximal polyposis of the stomach (GAPPS) was made from the phenotypic features of >100 gastric polyps, predominantly FGPs with antral/duodenal/colorectal sparing, a family history of dysplastic FGPs or gastric adenocarcinoma, and the genetic mutation in promoter 1B of the *APC* gene.^1^

**Figure 1:**
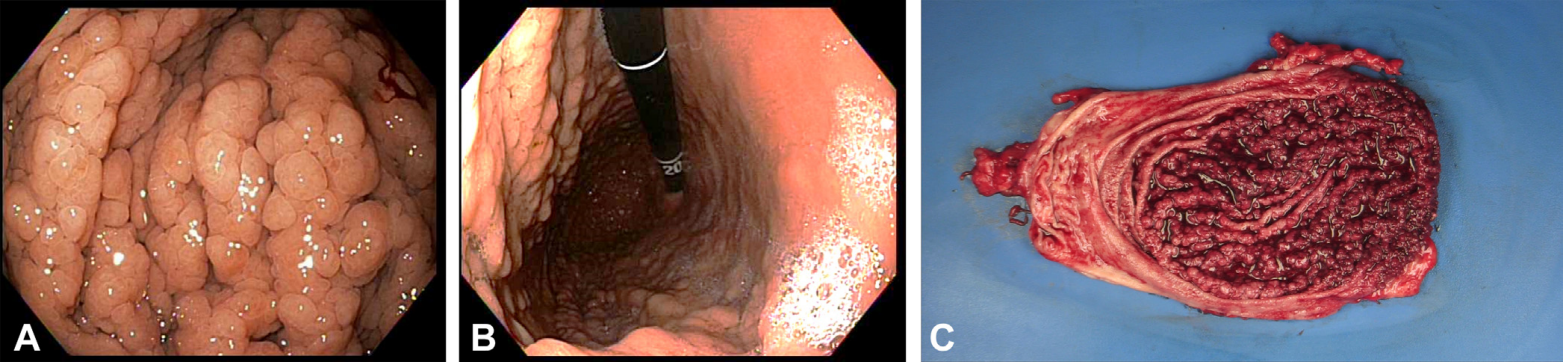
**A,** Esophagogastroduodenoscopy showing innumerable medium-sized sessile polyps in the gastric fundus. **B,** Esophagogastroduodenoscopy with the retroflexion view visualizing the fundus with polyps. There was complete antral and small bowel sparing. **C,** Laparoscopic total gastrectomy was performed. Dissection of the resected stomach confirmed the presence of innumerable polyps.

GAPPS has an autosomal dominant transmission with incomplete penetrance and was first recognized as a syndrome in 2012. It is one of the 3 hereditable syndromes that primarily cause gastric cancer, with the others being hereditary diffuse gastric cancer and familial intestinal gastric cancer.^2^ Unlike familial adenomatous polyposis (FAP), this variant is associated with a risk of gastric adenocarcinoma but not colorectal polyposis. Important differentials for conditions with FGPs needing to be ruled out are attenuated FAP, which may present with extracolonic manifestations, and the use of proton pump inhibitors.^3^^,^^4^

The rate of progression of GAPPS to gastric cancer is highly variable with several phenotypic variations, and ranges from 12% to 25%. Its management is individualized, and options include endoscopic surveillance with interval biopsies, understanding of its limitations, or risk-reduction surgical resection. Due to the inheritance pattern, genetic counseling is recommended; however, there is still a gap in the recommendations pertaining to genetic testing and screening among relatives. The first-degree relatives of the affected individual should consider endoscopy with biopsy sampling starting at the age of 15 years.^5^

Our patient underwent a prophylactic total gastrectomy. Pathology of the surgical specimen revealed no evidence of metaplasia, dysplasia, or malignancy (Fig. 1C). At the 6-month follow-up, she underwent an EGD that showed a well-healed esophagojejunostomy with complete resolution of symptoms. This case informs the pathognomonic endoscopic findings for the early recognition of this rare genetic cancer syndrome and the timely preventative management for prolonged survival.

## Patient Consent

The patient in this article has given informed consent for publication of their information and images.

## Disclosure

The following author disclosed financial relationships: D. Brauer: Consultant for Intera Oncology. All other authors disclosed no financial relationships. No funding sources supported this work.
